# ﻿Taxonomic notes on the genus *Itea* (Iteaceae)

**DOI:** 10.3897/phytokeys.239.117851

**Published:** 2024-03-12

**Authors:** Zhu-Qiu Song, Bu-Yun Zhang

**Affiliations:** 1 Key Laboratory of Plant Resources Conservation and Sustainable Utilization, South China Botanical Garden, Chinese Academy of Sciences, Guangzhou, China South China Botanical Garden, Chinese Academy of Sciences Guangzhou China; 2 South China National Botanical Garden, Guangzhou, China South China National Botanical Garden Guangzhou China

**Keywords:** China, lectotypification, synonym, taxonomy

## Abstract

The genus *Itea* (Iteaceae) is recognised as a genus with about 21 extant species of shrubs and trees. Within the genus, most species have oblong to elliptical leaves. The lanceolate and obolanceolate leaves are only found in three species, vix *Iteaamoena* Chun, *Iteariparia* Collett & Hemsl. and *Iteatenuinervia* S. Y. Liu. The results of our examination of literature, specimens and living plants in the wild have shown that *Iteatenuinervia* is conspecific with *Iteariparia* and is here reduced to a synonym of the latter species. The morphological description, colour pictures, voucher specimens, distribution map of *Iteariparia* and its related *Iteaamoena*, together with the morphological comparisons between the two species, are provided in this study.

## ﻿Introduction

*Itea* L. (Iteaceae) was first described based on the species *I.virginica* L. from eastern North America (Linnaeus 1753) and later it was reported in Asia, for example, *I.macrophylla* Wall. from tropical Asia (Wallich 1824), *I.nutans* Royle from western Himalaya (Royle 1835) and *I.japonica* Oliv. from Japan (Oliver 1865). *Itea* was previously divided into two sections based on its deciduous character, i.e. sect. Deciduae Engl. (= sect. Itea) and sect. Sempervirentes Engl. (Engler 1891). The former section comprises only two species with deciduous leaves, *I.virginica* and *I.japonica*, and the latter includes the other species with evergreen leaves. Recently, *Choristylisrhamnoides* Harv. (Harvey 1842) from southeastern Africa was transferred to *Itea* as the third section, i.e. sect. Choristylis (Harv.) Jordaan (Kubitzki 2007; Jordaan 2012). Thus, *Itea* was considered as an unusual genus with an East Asian-eastern North American-southeastern African disjunction pattern (Kubitzki 2007; Tian et al. 2021). The genus comprises about 21 extant species of shrubs and trees, including one in Africa, one in North America and 19 species in temperate, subtropical and tropical Asia (Tian et al. 2021; POWO 2023).

The leaf shapes of the genus *Itea* are usually oblong to elliptical, while the lanceolate and oblanceolate leaves only appear in three species, including *I.riparia* Collett & Hemsl. (Collett and Hemsley 1890), *I.amoena* Chun (Chun 1934) and *I.tenuinervia* S.Y. Liu (Liu 2001; Tian et al. 2021). *Iteariparia* was first published based on specimens collected on river-banks from Shan Hills, southern Shan States, Myanmar and it was characterised by narrow leaves, straight branches and erect racemes (Collett and Hemsley 1890). Gagnepain (1916) originally described *I.thorelii* based two gatherings from Vietnam and Laos and considered that it is very similar to *I.riparia*, but is different from the latter in the leaf width, the shape of anthers and the length of inflorescence, flower, stamen and ovary. Craib (1931) observed that some specimens of *I.riparia* from Thailand match *I.thorelii* in some respects. In China, *I.riparia* was recorded in Yunnan and *I.thorelii* was reported in Yunnan and Guangxi (Wu 1977; Fang and Qin 1985; Jin 1995), but the two species were merged in the Flora of China due to absence of obvious different characters (Jin and Ohba 2001). The earlier name *I.riparia* was adopted as its accepted name (Jin and Ohba 2001; Esser 2005; Chen and Liu 2015). The species grows on river-banks or near streams.

**Figure 1.**
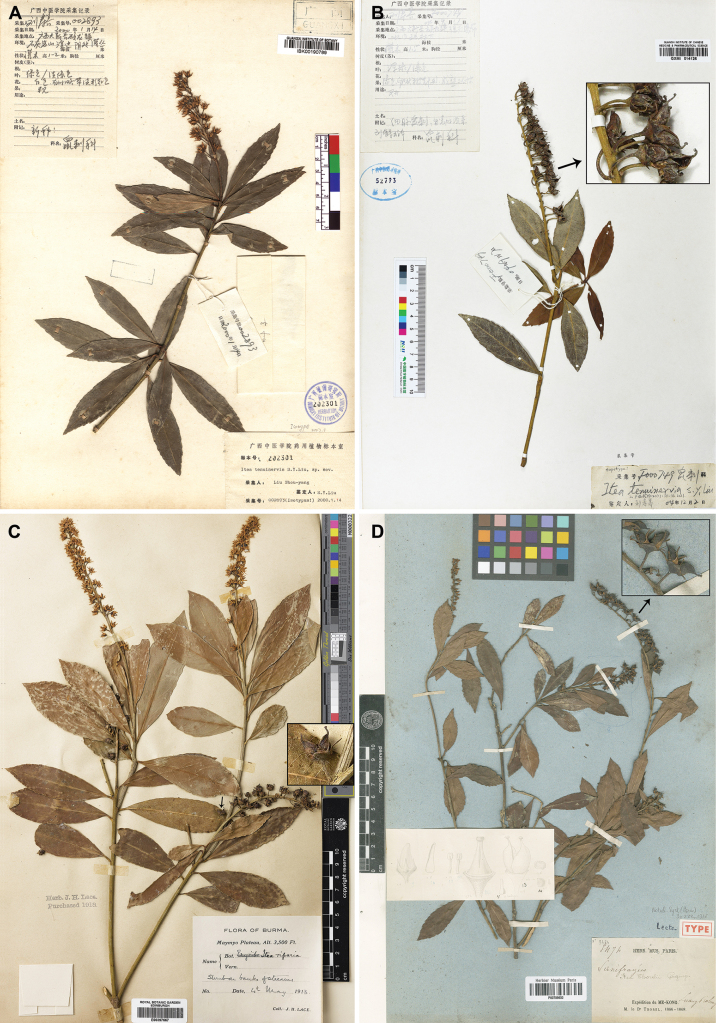
*Iteariparia***A** isotype of *Iteatenuinervia* (*Shou-Yang Liu 2893*, IBK00190789, https://www.cvh.ac.cn/spms/detail.php?id=c1cf1dd4) **B** topotype of *Iteatenuinervia* (*Shou-Yang Liu F000749*, GXMI014126), showing two divergent carpels per fruit **C** specimen of *Iteariparia* from Myanmar (*J. H. Lace s.n.*, E00397667, https://data.rbge.org.uk/herb/E00397667), showing two divergent carpels per fruit **D** lectotype of *Iteathorelii* (*Thorel 3474*, P00709632, https://science.mnhn.fr/institution/mnhn/collection/p/item/p00709632), showing two divergent carpels per fruit.

Chun (1934) described another *Itea* species with narrow leaves, *Iteaamoena*, based on specimens from southern Guangxi, China and noted that it is well characterised by narrow, long-acuminate leaves and by long lanceolate calyx lobes. The species was previously identified as “Itearipariavar.auminata Hu var. nov.”, based on *Ren-Chang Ching 8059* (Chun 1934) and recognised as “*Itealanceolata* Merr. n. sp.” based on *Kuan-Kwang Tsoong 1868* (Jin 1995). However, the latter two names have never been validly published. This species grows near streams, just like *I.riparia* (Fig. 5). Besides the habitat, *I.amoena* and *I.riparia* are also similar in the habit of evergreen shrubs, the lanceolate leaves and terminal inflorescences. Liu (2001) described *Iteatenuinervia* on the basis of a flowering collection found near streams in Shuolong Town, Daxin County, southwestern Guangxi, i.e. *Shou-Yang Liu 2893* (Fig. 1A) and it was morphologically compared with *I.riparia* and *I.thorelii* in the protologue. However, our examination of literature and specimens showed that there are no obvious differences between *I.tenuinervia* and *I.riparia* (including *I.thorelii*). Thus, this study treats *Iteatenuinervia* as a new synonym of *I.riparia* and discusses the morphological differences between *I.riparia* and *I.amoena*.

## ﻿Materials and methods

The specimens of the genus *Itea* kept in the Herbaria HITBC, IBSC and KUN have been examined by visiting these Herbaria and the images of *Itea* specimens deposited in the Herbaria E, GXMG, GXMI, IBK, IMDY, L, MW, N, NAS, NF, P, PE, SZ, TCD and US were also studied through the databases of specimens, such as Chinese Virtual Herbarium (CVH, https://www.cvh.ac.cn), Global Biodiversity Information Facility (GBIF, https://www.gbif.org), RBGE Herbarium Catalogue (https://data.rbge.org.uk/search/herbarium/), Naturalis BioPortal (https://bioportal.naturalis.nl/), Muséum National D'Histoire Naturelle (https://science.mnhn.fr/all/search) and Smithsonian National Museum of Natural History (https://collections.nmnh.si.edu/search/botany). Acronyms for the herbaria follow the Index Herbariorum (Thiers 2023). We also observed the living plants of the relevant species in the wild, including in the type localities of *I.tenuinervia* and *I.amoea*, as well as multiple localities of *I.riparia*.

## ﻿Taxonomic treatment

### ﻿1. Itea riparia

Taxon classificationPlantaeSaxifragalesIteaceae

Collett & Hemsl., J. Linn. Soc., Bot. 28: 57. 1890.

280D862C-A2D7-5919-B4B5-57DCFD731E38

Figs 1 , 2 , 3 , 4

= Iteathorelii Gagnep., Notul. Syst. (Paris) 3: 222.1916. Type. Laos. Luang-prabang, 1866–1868, *Thorel 3474* (lectotype: P00709632, photo!, designated by Lecompte 1965: 40; isolectotypes: P00709633 photo!, P00709634 photo!).
= Iteatenuinervia S. Y. Liu, Guihaia 21(1): 35. 2001. Type. China. Guangxi Province, Daxin County, Shuolong Town, by stream, 14 Jan 2000, *Shou-Yang Liu 2893* (holotype: GXCM; isotype: IBK00190789!), syn. nov.

#### Type.

Myanmar. Southern Shan States, Shan hills, on river-banks, 2000–4000 feet (ca. 700–1400 m) elev., *H. Collett s.n.* (holotype, K).

#### Description.

Shrub, evergreen, usually 0.5–2 m tall, sometimes up to 7 m. Stipules subulate, 0.2–0.7 mm long, caducous. Leaves alternate, both surfaces glabrous, chartaceous, lanceolate or oblanceolate, 3.5–13 cm long, 1–5 cm wide, length/width ratio is 2.6–5.2 (mean = 3.6), acute or slightly acuminate at apex, cuneate at base, distantly shallowly subglandular crenate-serrate along upper margin; mid-vein raised on both surfaces, lateral veins in 4–7 pairs, arcuate ascending, lateral and reticulate veins visible and impressed above surface; petiole 0.4–1.2 cm long, raised above. Pseudoracemes, terminal or sometimes also axillary, usually solitary, sometimes with short branches at base, 4–18 cm long, rachis puberulous, usually 2- or 3-flowered per node; bracts at base of pedicel, subulate, 2–3 mm long, 0.3–0.5 mm wide, deciduous. Flowers white; pedicel erect, puberulous, 3–11 mm long; calyx shallowly cupular; calyx lobes 5, triangular, green, 1.5–2 mm long, 1.2–1.5 mm wide at base, puberulous; petals 5, white, triangular-lanceolate, acute at apex, widest at base, ca. 5 mm long, 1.5–1.9 mm wide at base, glabrous, erect-spreading at anthesis, becoming green and thickened at fruiting; stamens 5, alternating with the petals, shorter than petals, 3.3–3.6 mm long; filaments glabrous, ca. 0.8 mm wide; anthers oblong, ca. 0.6 mm long, 0.4 mm wide; floral disc annular, fleshy, slightly yellow; ovary semi-inferior, consisting of two carpels diverging in the middle and united at the top; stigma capitate. Capsule glabrous, 5–8 mm long, consisting of two obviously divergent carpels, with persistent sepals and petals, turning brown and dehiscing along the ventral suture at mature. Seed numerous, white at immature and brown at mature, 1–1.7 mm across; testa slightly granular.

#### Distribution.

The species is distributed in China, Laos, Myanmar, Thailand and Vietnam (Fig. 6). It usually occurs near streams under forests, with elevation range from 200 to 1400 m.

#### Specimens examined.

**China.** Guangxi: Daxin, 15 May 2008, *Guangxi Exped. Inst. Bot. 498* (IBK00278403), 17 Sept 2004, *Shou-Yang Liu F000749* (GXMI014126), 400 m elev., 9 Jun 2023, 22.854261°N, 106.725634°E, *Zhu-Qiu Song et al. JZ20230549* (IBSC); Jingxi, 457 m elev., 30 June 2013, *En-De Liu 3996* (KUN1241727), 500 m elev., 10 May 2023, 22.991658°N, 106.674116°E, *Bu-Yun Zhang et al. JZ20230371* (IBSC), 500 m elev., 9 Jun 2023, 23.009193°N, 106.659319°E, *Zhu-Qiu Song et al. JZ20230553* (IBSC); Napo, 450 m elev., 18 May 1943, *Pu-Chin Tsoong & Ko-Zen Kuang 356* (PE00865551, PE00865552), 15 Apr 1977, *Yuan Lin et al. 3-5104* (GXMI014127, GXMI014128). Yunnan: Cangyuan, 700 m elev., 29 May 1974, *Yan-Hui Li 11737* (HITBC015745, HITBC015750, IBSC0273493, KUN0477791, KUN0540565, KUN0540567), 790 m elev., 15 May 1979, *Yan-Hui Li 20907* (HITBC015744, HITBC015751, KUN0540559, KUN0540568); Jinghong, 540 m elev., 7 May 1955, *Kuo-Mei Feng 20754* (IBSC0273491, KUN0477788, KUN0477789, PE00865549, SZ00179768), 30 Aug 2019, *Yun-Hong Tan ML56* (XSBN002276); Malipo, 1100 m elev., 21 Jun 1940, *Chi-Wu Wang 86321* (KUN0477792, PE00865553), 1000 m elev., 3 Feb 1940, *Chi-Wu Wang 86599* (KUN0477790); Mengla, 17 Jan 1976, *Lai-Yun Xiao 11269* (HITBC099861, IBSC0273492), 680 m elev., 18 Dec 1986, *Shao-Rong Guo 592* (IMDY0008066), 850 m elev., 11 Feb 1960, *Yan-Hui Li 2813* (HITBC015743, IBK00170997, IBSC0273495, KUN0477787), 560 m elev., 14 Apr 1961, *Yan-Hui Li 3049* (HITBC015746), 11 May 1961, *Yan-Hui Li 3222* (HITBC015747, IBSC0273494, KUN0477785), Jun 1967, *Yunnan First Section 138* (PE00865550), 600 m elev., 2 Sept 2023, 21.989364°N, 101.368001°E, *Zhu-Qiu Song et al. JZ20231022* (IBSC); Zhenkang, 12 Apr 1976, *Yan-Hui Li 20116* (HITBC015748, HITBC015749, KUN0477786). **Laos.** Champasak: Paksong, 29 Apr 2013, H*ang Sun et al. sunhang16056* (KUN1256568, KUN1256569); Luang Namtha: Nateuy, 1130 m elev., 21 Apr 2006, *En-De Liu 1686* (KUN1204594); Luang Prabang: 1866–1868, *Thorel 3474* (P00709632, P00709633, P00709634); Phongsal: 30 Apr 1993, *Guo-Da Tao et al. 930162* (HITBC059236, HITBC059244); Vientiane: Mt. Phu Kao Kwai, 700–800 m elev., 31 Dec 1993, *N. Fukuoka & H. Koyama L-65039* (L4134635); Without precise locality, *H. Bon s.n.* (P03179260). **Myanmar.** Maymyo: 760 m elev., 21 Apr 1916, *A. Rodger 510* (NAS00337458), 1066 m elev., 8 May 1912, *J. H. Lace 5802* (E00397666), 1066 m elev., 4 May 1912, *J. H. Lace s.n.* (E00397667); Taunggyi: 1219 m elev., Apr 1939, *F. G. Dickason 8273* (E01033109). **Thailand.** Chaiyaphum: Phu Khieo, 600–700 m elev., 1972, *K. Larsen et al. 31288* (L1861211), Ban Nam Phrom, 600 m elev., 24 May 1974, *R. Geesink et al. 6886* (L1861215, P00392452); Chiang Mai: Thawatchai, Kasem, 700 m elev., 7 May 1991, *Guo-Da Tao et al. 653* (KUN0540552), Me Ka Pak Bank, 1300 m elev., 21 Apr 1928, *H. B. Garret 505* (L1861224, L1861225, P03179261), Mae Rim District, 600 m elev., 12 Jun 2009, *J. F. Maxwell 09-162* (L2058714, P00911865), Doi Sutep, Mae Sa Falls, 10 Apr 1989, *J. F. Maxwell 89-446* (L1861217), Doi Intson National Park, 650 m elev., 4 May 1997, *J. F. Maxwell 97-443* (L4160466), Doi Inthanon, 1400 m elev., 3 Jan 1975, *R. Geesink et al. 8048* (L1861214, P03371228), Doi Inthanon, 920–960 m elev., 21 Jul 1988, *S. Tsugaru T-61686* (L4161221), 18 Aug 1970, *Voravut 8* (L1861218); Kanchanaburi: Sangklaburi, Toong Yai Narssuan Wildlife Reserve, 200 m elev., 12 Jan 1994, *J. F. Maxwell 94-10* (L4134592), near Khwae Noi River, Pompee Village, 250 m elev., 25 Mar 1968, *C. F. van Beusekom & C. Phengkhlai 108* (L1861220), 800 m elev., 30 Apr 2004, *M. van de Bult 796* (L4160559); Nakhon Ratchasima: Khao Yai, 800 m elev., 25 Mar 1968, *B. Hansen et al. 11340* (L1861219, L1861221); Phrae: Mae Bhaem stream, 440 m elev., 5 Jan 1972, *C. F. van Beusekom et al. 4661* (L1861223); Tak: Pha Charoen Waterfall National Park, 20 June 2005, *Pooma et al. 5329* (L4160075), Lansang National Park, 600 m elev., 21 Jul 1973, *G. Murata et al. 16617* (L1861212, P03179258), Lan Sang National Park, 250–400 m elev., 30 Aug 1967, *M. Tagawa et al. 8558* (L1861213), Larn Sarng Nat. Park, 350 m elev., 29 May 1973, *R. Geesink et al. 5532* (L1861222, P03179259); Without precise locality, Apr 1921, *A. F. G. Kerr 5273* (TCD0016587), Mar 1924, *A. F. G. Kerr 8763* (TCD0016588). **Vietnam.** Dak Lak: Lak, Chu Yang Sin National Park, 900 m elev., 23 Mar 2013, *M.S. Nuraliev 724* (MW0738997); Hoa Binh: Yen Thuy, Cuc Phuong National Park, 12 Sept 2002, *M. V. Xinh MVX 241* (L1861216), Barrage de Cho-bo (Rivière Noire), sur les roches calcaires atteintes par les hautes eaux, 18 Oct 1888, *Balansa 3152* (P00709635).

#### Taxonomic notes.

**Figure 2.**
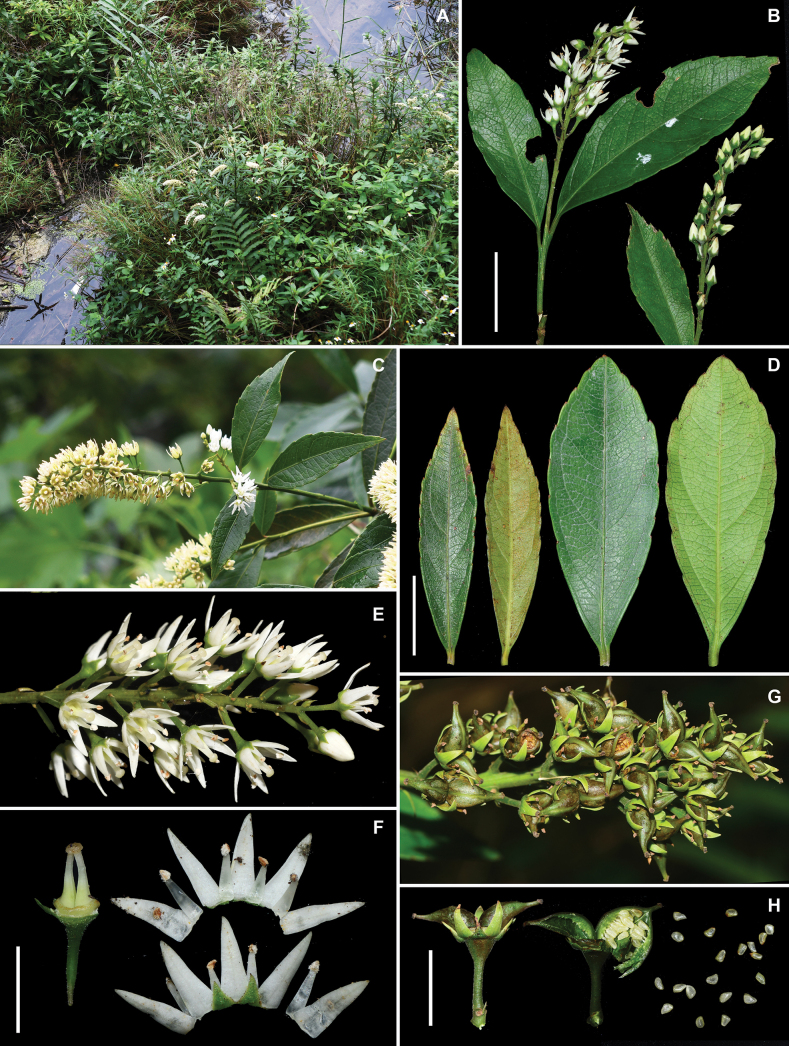
*Iteariparia* from Jingxi County, Guangxi, China **A** habitat **B–C** terminal inflorescences **D** leaves in adaxial and abaxial views **E** inflorescences **F** flowers with the different parts separated **G** infructescence, showing two divergent carpels per fruit and persistent sepals and petals **H** fruits and seeds. Voucher specimens: *Bu-Yun Zhang et al. JZ20230371* (fl., IBSC), *Zhu-Qiu Song et al. JZ20230553* (fr., IBSC). Scale bars: 2 cm (**B, D**); 5 mm (**F**); 1 cm (**H**).

**Figure 3.**
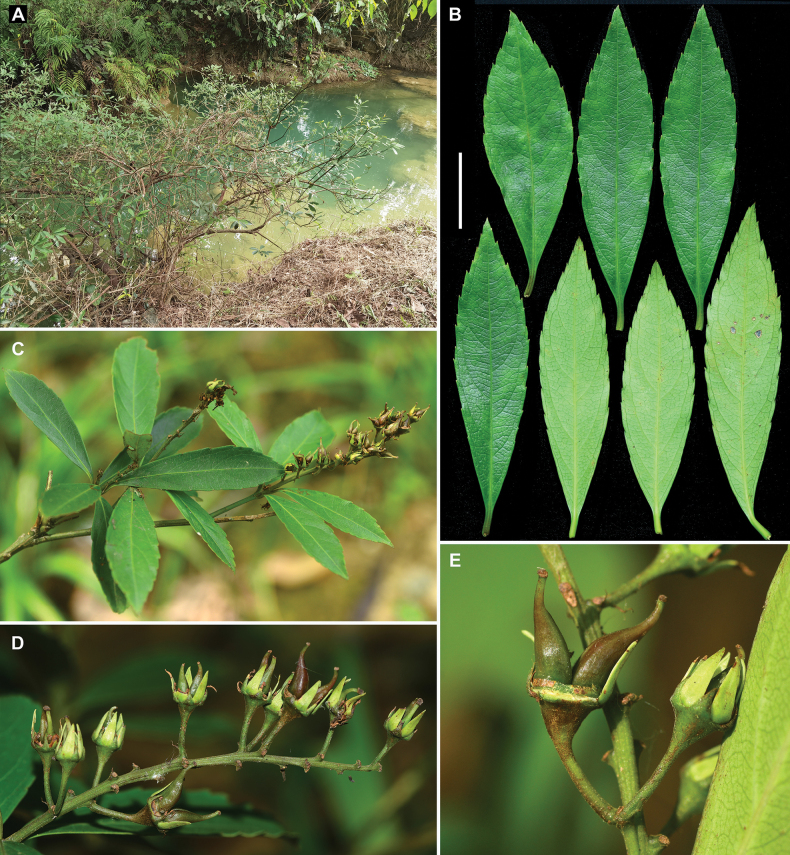
*Iteariparia* from Daxin County, Guangxi, China **A** habitat **B** leaves in adaxial and abaxial views **C** fruiting branchlets **D** infructescence **E** fruits, showing two divergent carpels per fruit and becoming green and thickened petals and persistent sepals and petals. Voucher specimens: *Zhu-Qiu Song et al. JZ20230549* (IBSC). Scale bar: 2 cm (**B**).

**Figure 4.**
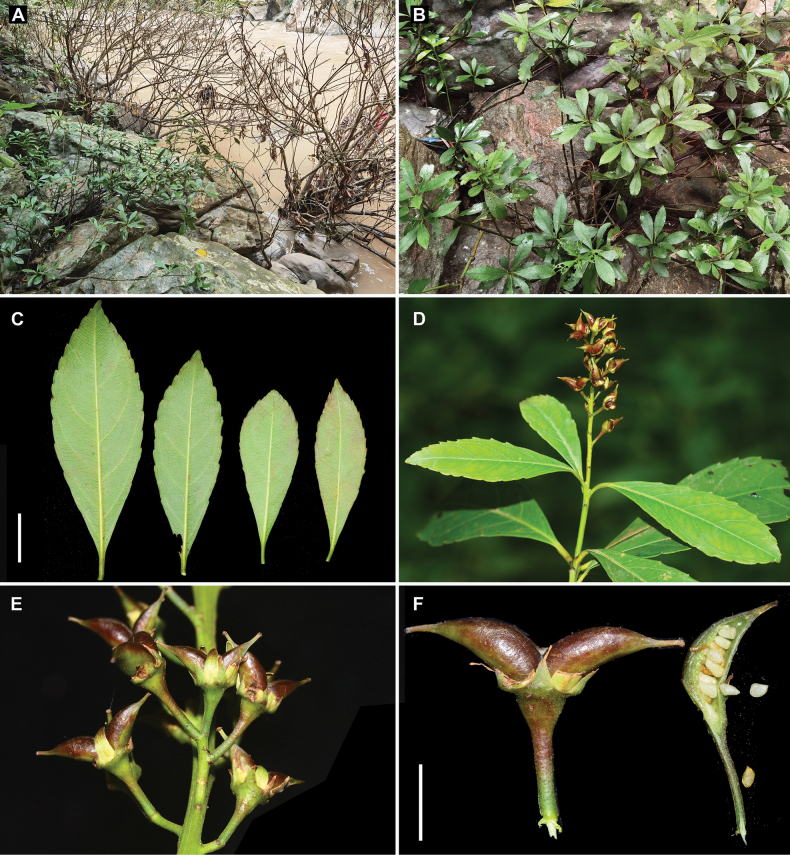
*Iteariparia* from Mengla County, Yunnan, China **A** habitat **B** habit **C** leaves **D** fruiting branchlet **E** part of infructescence **F** fruits and seeds, showing two divergent carpels per fruit. Voucher specimens: *Zhu-Qiu Song et al. JZ20231022* (IBSC). Scale bars: 2 cm (**C**); 5 mm (**F**).

In the protologue, Liu (2001) considered that *I.tenuinervia* is distinguished from *I.riparia* in the height (1–2 m vs. 6 m), leaf shape (long elliptical, rarely oblanceolate vs. oblong, elliptical to lanceolate), pedicel length (10 mm vs. 3–5 mm) and petal length (6 mm vs. 4 mm) and it is distinguished from *I.thorelii* in the leaf shape (long elliptical, rarely oblanceolate vs. oblanceolate), inflorescence type (racemes vs. racemose panicles), bract shape (triangular-lanceolate vs. linear-lanceolate), petal shape (narrowly lanceolate var. oblong-triangular) and pedicel length (10 mm vs. 4–6 mm). Subsequently, the species was accepted by Chen and Liu (2015). However, our examination of literature and specimens showed that there are no obvious differences between *I.tenuinervia* and *I.riparia* (including *I.thorelii*) in the characters above (see Table 1, Fig. 1). In the protologue, Liu (2001) did not describe the fruits of *I.tenuinervia*, but he collected a fruiting gathering at the type locality in 2004, i.e. *Shou-Yang Liu F000749*, which shows two obviously divergent carpels per fruit (Fig. 1B). The fruits with two obviously divergent carpels are very special in the genus *Itea*, because most species bear two partly or completely fused carpels before dehiscence (Jordaan 2012) and only *Iteariparia* (including *I.thorelii*) was found to have two obviously divergent carpels (see Lecompte (1965: Pl. IV 8–10); Wu (1977: Pl. 26)). Thus, in the fruit character, *I.tenuinervia* is very similar to *I.riparia* as well. Furthermore, our observations of living plants in the wild, including in the type locality of *I.tenuinervia*, also showed that *I.tenuinervia* is consistent with *I.riparia* in the morphological characters and habit (Figs 2–4).

**Table 1.** Comparison between *Iteatenuinervia* and *Iteariparia*.

Characters	*Iteatenuinervia*	*Iteariparia*
Habit	shrubs 1–2 m	shrubs 1–6 m tall
Branchlet	green, striate, glabrous	yellow-green, striate, glabrous
Leaf arrangement	alternate, usually crowded at upper and lower parts of branchlets	alternate, usually crowded at apex of branchlets, subclustered
Leaf texture	thinly leathery	thinly leathery
Leaf hairiness	glabrous	both surfaces glabrous
Leaf shape	long elliptic, rarely oblanceolate, base cuneate, apex acuminate, margin incurved glandular dentate	elliptic to lanceolate or obovate-elliptic, base cuneate, apex acute or acuminate, margin curved cartilaginous glandular dentate
Leaf size	5–9 × 1.7–2.2 cm	5–13 × 1.5–5 cm
Lateral veins	4–5 pairs, confluent near margin, raised abaxially, slightly sunken adaxially	4–7 pairs, arcuate, confluent near margin, slightly raised abaxially, obscure adaxially
Tertiary veins	invisible above and visible below	reticulate, invisible above
Petiole	5 mm, glabrous	4–12 mm, glabrous
Stipules	ca. 1.5 mm long, caducous	0.2–0.7 mm long
Inflorescence	racemes terminal, 8–14 cm; rachis puberulous; flowers numerous, rather crowded, often 2–3-clustered	racemes terminal, 4–18 cm; rachis puberulous; flowers numerous, rather crowded, often 3-clustered
Bracts and bracteoles	bracts triangular-lanceolate, 3–5 mm; bracteoles 1–1.5 mm long	bracts ca. 3 by 0.5 mm, caducous, bracteoles to 1 mm long, similar
Pedicels	(6–) 10 mm	3–6 mm, puberulous
Calyx	shallowly cupular; lobes triangular, ca. 2 mm	shallowly cupular; lobes erect at anthesis, triangular-lanceolate, 1.5–4 mm
Petals	5, white, sometimes slightly pink, narrowly lanceolate, ca. 6 mm long, erect at anthesis	5, white, distinctly larger than the sepals, 4–5 by 1.5–2 mm, erect
Stamens	5, situated opposite the calyx lobes; filaments 5 mm long, slightly wider below the middle, glabrous; anthers ovoid, dorsifixed.	5, situated opposite the calyx lobes; filaments 2.5–3 mm long, glabrous; anthers ca. 1 mm long, dorsifixed, ovoid.
Disc	Ring-like, yellow	Ring-like
Ovary	semi-inferior, consisting of two carpels diverging above the base at the end of flowering, with a capitate stigma	superior or semi-inferior, glabrous, consisting of two carpels diverging in the middle and united at the top in a short style, surmounted by a capitate stigma
Fruit	capsules ovoid-conical, with very divergent carpels, except at the base; dehiscence by a slit corresponding to the ventral suture (based on a topotype, *Shou-Yang Liu F00074*9, Fig. 1B)	capsules ovoid-conical, ca. 5 mm, glabrous, with very divergent carpels, except at the base; dehiscence by a slit corresponding to the ventral suture
Phenology	Fl. Jan., fr. Sept.	Fl. and fr. May–Feb.
Distribution	Guangxi, China	Myanmar, Thailand, Laos, Vietnam, China (Guangxi, Yunnan)
References	Liu (2001)	Lecompte (1965), Jin and Ohba (2001), Esser (2005)

### ﻿2. Itea amoena

Taxon classificationPlantaeSaxifragalesIteaceae

Chun, Sunyatsenia 1(4): 238. 1934.

D68E708C-BC0C-522D-AB43-64F73FD60604

Fig. 5

#### Type.

China. Guangxi Province, Shangsi County, Shup-man-tai Shan [Shi Wan Da Shan], in shaded ravine, about 1200 ft. (ca. 400 m), 29 Jul 1933, *Ching-Lieh Tso 23439* (lectotype, designated here: IBSC0004326, isolectotypes: IBSC0004324, IBSC0004325, PE00864804).

#### Description.

Shrub, evergreen, usually 0.2–0.5 m, sometimes up to 2 m tall, glabrous, except puberulent on inflorescences. Stipules subulate, ca. 2 mm long, caducous. Leaves alternate, both surfaces glabrous, thinly leathery, narrowly lanceolate, 6.5–13.9 cm long, 1.2–2.2 cm wide, length/width ratio is 4.1–9.5 (mean = 6.5), acuminate or gradually acute at apex, cuneate or subobtuse at base, distantly shallowly subglandular crenate-serrate along upper margin; mid-vein raised on both surfaces, lateral veins in 6–8 pairs, arcuate ascending, lateral and reticulate veins obviously visible and prominently elevated above surface; petiole 1–1.5 cm long, narrowly grooved above. Pseudoracemes, terminal or sometimes also axillary, solitary, 6–24 cm long, rachis puberulous, usually 2- or 3-flowered per node; bracts at base of pedicel, subulate, ca. 1–2 mm long, deciduous. Flowers white; pedicel erect, puberulous, 5–9 mm long; calyx shallowly cupular; calyx lobes 5, narrowly lanceolate, green, 2–2.5 mm long, 0.8–1 mm wide at base, puberulous; petals 5, white, reflexed at anthesis, deciduous at fruiting, triangular-lanceolate, acute at apex, widest at base, ca. 5 mm long, ca. 1.5 mm wide at base, glabrous; stamens 5, alternating with the petals, shorter than petals, ca. 4 mm long; filaments glabrous, ca. 0.3–0.4 mm wide; anthers oblong, ca. 0.5 mm long, 0.3 mm wide; floral disc annular, fleshy, slightly yellow; ovary semi-inferior, narrowly grooved, consisting of two united carpels; stigma capitate. Capsule suboblong-conical, 5–6 mm, glabrous, turning brown and dehiscing septicidally at mature, with persistent sepals. Seed numerous.

#### Distribution.

The species is only found in Guangxi, southern China (Fig. 6). It usually occurs near streams under evergreen forests, with elevation range from 90 to 800 m.

#### Specimens examined.

China. Guangxi: Dongxing City, Malu, 107°58'56"E, 21°41'8"N, 90 m elev., 12 Oct 2018, *Dongxing Exped. 450681181012069LY* (GXMG0206492, IBK00418425), 21 Jun 1919, *Kuan-Kwang Tsoong 1868* (PE00864807), 26 Sept 1973, *Chou-Fen Liang 33611* (IBK00170636); Fangchenggang City, Pingwang, 15 Jul 2007, *Feng-Juan Mou 127* (IBSC0712193), Banba, 15 Oct 1973, *Chou-Fen Liang 33875* (IBK00170634), Nangui, 11 Nov 1958, *Chao-Chien Chang* 13197 (IBK00170635, IBSC0272608), Naliang, 24–31 Jul 1936, *Wai-Tak Tsang 26510* (IBSC0272609, P03179693), Naliang, 105 m elev., 13 Apr 1956, *Hepu Pl. Exped. 2365* (IBSC0272605, PE00864802), Naliang, 107°47.563'E, 21°47.218'N, 140 m elev., 13 Jan 2016, *Pu Zou & Kai Xu zp57* (IBSC0824452); Nalungou, 350 m elev., 21 Jul 1982, *Peng-Cheng Huang 216* (NF2011745, NF2011746); Shangsi County, Shi Wan Da Shan, 325 m elev., 9 Nov 1958, *Chao-Chien Chang* 12233 (IBSC0272601), Wangle, 107°49'36"E, 21°53'24"N, 221 m elev., 1 Apr 2009, *Wei-Bin Xu et al. liuyan0208* (KUN0906334, KUN0902457), Shi Wan Da Shan, 8 Jul 1937, *H. Y. Liang 69500* (IBK00170632, IBK00170633, IBSC0272602, PE00864803), Shi Wan Da Shan, 11–30 Jul 1934, *Wai-Tak Tsang 23804* (IBSC0272603), 15–24 Aug 1936, *Wai-Tak Tsang* 26675 (IBSC0272604, P03179692), Seh-feng Dar Shan [Shi Wan Da Shan], 2500 feet (ca. 800 m) elev., 21 Oct 1928, *Ren-Chang Ching* 8059 (IBSC0272600, N117066318, PE00864805, PE00864806, US03684023), 250 m elev., 14 Aug 1986, *Beijing Youth Exped. 718* (PE01869678), 90 m elev., 23 Dec 1943, *S. H. Chun 3304* (IBSC0272607), 170–300 m elev., 24 May 1944, *Shao-Hing Chun* 5209 (IBSC0272606), 140 m elev., 8 Jun 2023, 21.097495°N, 108.257582°E, *Zhu-Qiu Song et al. JZ20230531* (IBSC), 320 m elev., 8 Jun 2023, 21.882874°N, 107.8346332°E, *Zhu-Qiu Song et al. JZ20230537* (IBSC).

**Figure 5.**
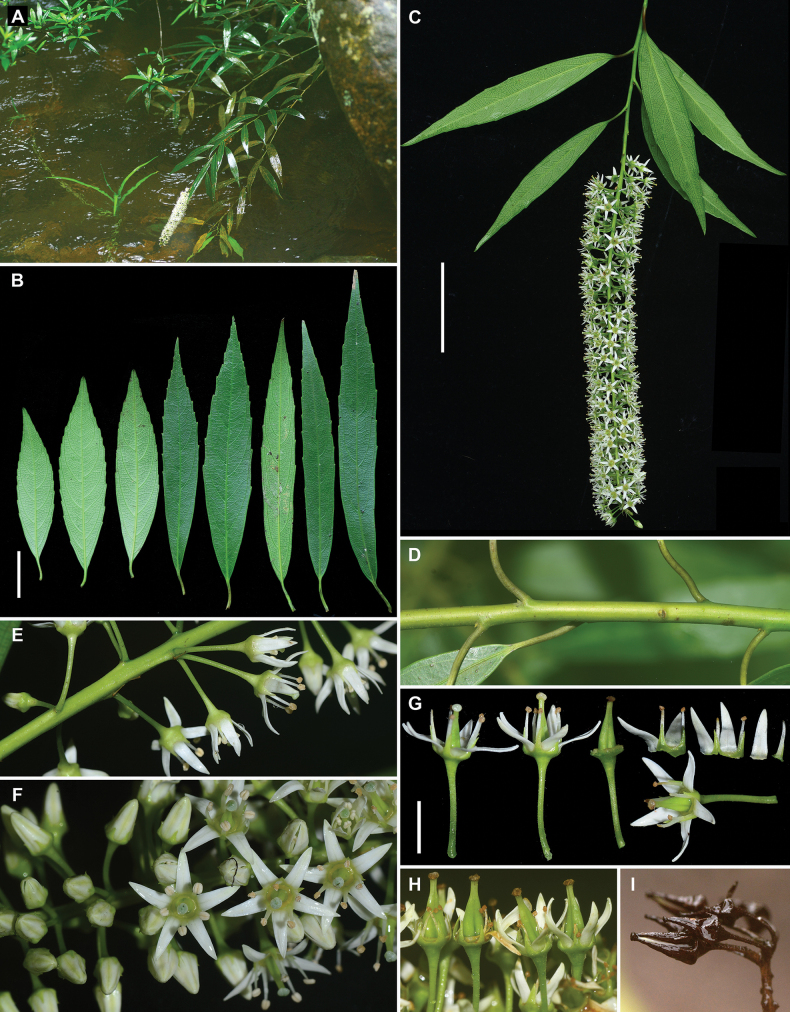
*Iteaamoena* from Shangsi County, Guangxi, China **A** habitat **B** leaves in adaxial and abaxial views **C** flowering branchlet **D** branchlet **E** part of inflorescences, showing usually 2 or 3 flowers per node **F** part of inflorescences, showing floral petals reflexed at anthesis **G** flowers with the different parts separated **H** flowers at the end of flowering, showing two fused carpels per fruit and deciduous petals **I** mature fruits. Voucher specimens: *Zhu-Qiu Song et al. JZ20230531*, *JZ20230537* (IBSC). Scale bars: 2 cm (**B**); 4 cm (**C**); 5 mm (**G**).

#### Taxonomic notes.

**Figure 6.**
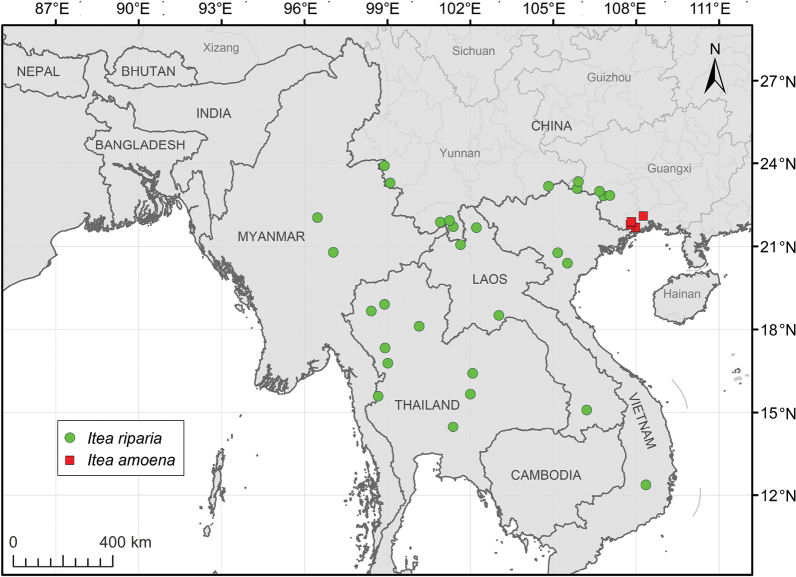
Distribution map of *Iteariparia* (green circles) and *Iteaamoena* (red squares).

*Iteaamoena* can be readily distinguished from *I.riparia* in its long narrower leaves (length: width = 4.1–9.5 and ratio mean = 6.5 vs. 2.6–5.2 and 3.6), raised lateral and reticulate veins in the adaxial leaf surface (vs. impressed veins), floral petals reflexed at anthesis (vs. floral petals erect at anthesis), floral petals deciduous at fruiting (vs. becoming green and thickened at fruiting), lanceolate calyx lobes (vs. triangular calyx lobes) and fruits with two fused carpels (vs. two obviously divergent carpels). In distribution, *I.amoena* is restricted to southern Guangxi and it does not overlap with *I.riparia* (Fig. 6).

## Supplementary Material

XML Treatment for Itea riparia

XML Treatment for Itea amoena

## ﻿Additional information

### Author ORCIDs

Zhu-Qiu Song https://orcid.org/0000-0002-7763-2431

Bu-Yun Zhang https://orcid.org/0000-0003-1201-7638

### Data availability

All of the data that support the findings of this study are available in the main text.
